# Resveratrol Treats UVB-Induced Photoaging by Anti-MMP Expression, through Anti-Inflammatory, Antioxidant, and Antiapoptotic Properties, and Treats Photoaging by Upregulating VEGF-B Expression

**DOI:** 10.1155/2022/6037303

**Published:** 2022-01-04

**Authors:** Baining Cui, Yan Wang, Jiahui Jin, Zhen Yang, Ruoxi Guo, Xue Li, Lei Yang, Zheng Li

**Affiliations:** ^1^College of Pharmaceutical Engineering of Traditional Chinese Medicine, Tianjin University of Traditional Chinese Medicine, Tianjin 301617, China; ^2^Chinese Materia Medica College, Tianjin University of Traditional Chinese Medicine, Tianjin 301617, China; ^3^Joint Laboratory for Research on Components of Traditional Chinese Medicine Cosmetics Established by Tianjin University of Traditional Chinese Medicine and Tianjin ShangMei Cosmetics Co., Ltd., Tianjin ShangMei Cosmetics Co., Ltd., Tianjin 300380, China; ^4^Department of Nuclear Medicine, Tianjin Medical University General Hospital, Tianjin 300052, China; ^5^Tianjin Institute of Acute Abdominal Disease of Integrated Traditional Chinese and Western Medicine, Tianjin 300100, China

## Abstract

UVB exposure is one of the primary factors responsible for the development of photoaging, and the aim of this study was to investigate the mechanism involved in the photoprotective properties of resveratrol (RES) in UVB-induced photoaging. Photoaging models of Hacat cells and ICR mice were established by UVB irradiation. The effect of RES on cell viability was then assessed using the MTT assay. The effect of RES on reactive oxygen species (ROS) production was detected through a fluorescent probe assay. The effect of RES on oxidized glutathione (GSSH) content, and superoxide dismutase (SOD) activity in photoaging Hacat cells, were measured separately, using kits. An enzyme-linked immunosorbent assay (ELISA) was used to measure the effect of RES on IL-6 secretion. The effect of VEGF-B on RES photoprotection was examined through the RT-qPCR method, after silencing VEGF-B through siRNA transfection. For animal experiments, the relative water content of the skin of ICR mice was determined using the Corneometer CM825 skin moisture tester. Starting from the third week of the study, the back skin of photoaging ICR mice was photographed weekly using the TIVI700 camera, and the depth of skin wrinkles in photoaging ICR mice was also analyzed. The thickness of the epidermis in photoaging ICR mice was assessed by the hematoxylin-eosin (HE) staining method. The content of collagen fibers in the skin dermis of photoaging ICR mice was measured by the Masson trichrome staining method. The content of collagen III in the dermis of the skin in photoaging ICR mice was measured through immunohistochemistry (IHC) techniques. The effect of RES on the mRNA expression levels of MMP-1, MMP-9, HO-1, GPX-4, IL-6, TNF-*α*, VEGF-B, caspase9, and caspase3 in photoaging Hacat cells, and that of MMP-3, Nrf2, HO-1, NQO1, SOD1, GPX-4, caspase9, caspase3, and IL-6 in the skin of photoaging ICR mice, was measured by RT-qPCR. The effects of RES on caspase3, Nrf2 (intranuclear), COX-2, P-ERK1/2, ERK1/2, P-P38MAPK, and P38MAPK in photoaging Hacat cells, and on MMP-9, caspase3, COX-2, P-JNK, P-ERK1/2, and P-P38MAPK protein expression in the skin of photoaging ICR mice, were assayed by the WB method. The results of this study, therefore, show that RES has a protective effect against UVB-induced photoaging in both Hacat cells and ICR mice. Its mechanism of action may include reducing the expression of MMPs and the secretion of collagen and inflammatory factors by inhibiting the ROS-mediated MAPK and COX-2 signaling pathways, balancing oxidative stress in the skin of Hacat cells and ICR mice by promoting the Nrf2 signaling pathway, inducing antiapoptotic effects by inhibiting caspase activation, and exerting antioxidant and antiapoptotic effects by targeting the VEGF-B, demonstrating its photoprotective effects against UVB irradiation-induced photoaging.

## 1. Introduction

Photoaging accounts for approximately 80% of skin aging [1]. One of the main causes of photoaging of the skin is UV radiation. Based on the wavelength, ultraviolet light is classified into UVA (315-400 nm), UVB (280-315 nm), and UVC (200-280 nm) radiation. Of these, UVC radiation is almost entirely absorbed by the ozone layer, and UVB has greater genotoxicity and capacity for sunburn, as compared to UVA radiation [2, 3]. Prolonged exposure of the skin to UVB can lead to erythema, sunburn, oxidative damage and inflammation, and sometimes even skin cancer [4–6].

The primary mechanisms associated with UVB-induced skin photoaging include causing oxidative stress [7], stimulating an inflammatory response [8], abnormal expression of matrix metalloproteinases (MMPs) [9], and so on. Excessive UVB irradiation of skin cells can lead to a large accumulation of intracellular ROS [10]. Excess ROS can cause an inflammatory cascade of skin photoaging, such as photoaging induced by NF-*κ*B-TNF-*α*-mediated inflammatory pathways [11]. Moreover, ROS is vital to the regulation of collagen metabolism and can lead to increased expression of at least three MMPs in the human skin, namely, MMP-1, MMP-3, and MMP-9 [12–14]. On the other hand, ROS is considered to initiate the activation of the MAPK signaling pathway. MAPK is a family of serine and tyrosine kinases containing extracellular signal-regulated kinase (ERK), c-Jun N-terminal kinase (JNK), and p38 mitogen-activated protein kinase (p38 MAPK), that are involved in the regulation of cell proliferation, differentiation, apoptosis, and inflammation. The p38MAPK pathway is regulated through transcriptional and posttranscriptional mechanisms to affect cell death signaling as well as proapoptotic (Bax) and antiapoptotic (Bcl-2). Subsequently, the cystatin protease signaling pathway (caspase3) is altered as well, leading to apoptosis [15]. Therefore, targeting MAPK-regulated signaling pathways could potentially be an effective method in countering UVB-induced apoptosis.

Nuclear factor-erythroid 2-related factor 2 (Nrf2) protects skin cells from UV radiation-induced oxidative damage and cellular dysfunction [16]. The Nrf2/antioxidant response element (ARE) pathway is considered to be one of the principal defense mechanisms against oxidative stress, regulating the expression of multiple detoxification/antioxidant genes, such as heme oxygenase-1 (HO-1), quinone oxidoreductase 1 (NQO1), CAT, and SOD [17, 18]. When induced by antioxidants, Nrf2 disrupts Kelch-like ECH-associated protein 1 (Keap1), the primary molecule responsible for its negative regulation, which is followed by rapid nuclear translocation and transactivation of ARE-associated genes [19]. This process promotes ROS scavenging, maintains redox homeostasis, suppresses inflammation, and repairs damaged DNA, thereby supporting the survival of cells in a pro-oxidant environment [20].

Vascular endothelial growth factor (VEGF) is a highly specific provascular endothelial growth factor that includes VEGF-A, VEGF-B, VEGF-C, VEGF-D, and placental growth factor [21]. Of these, VEGF-B plays a limited role in the development of vascular endothelial cells and in the growth of blood vessels. Nevertheless, further research showed that VEGF-B exhibited antioxidant [22], anti-inflammatory [23], and antitumour [24] properties, but there is limited research on the effects of VEGF-B on photoaging.

Resveratrol (RES), also known as 3,4′,5-trihydroxystilbene, is a polyphenolic plant antitoxin synthesized in response to fungal infections and was originally identified from the roots of the hairy quinoa plant. Recent research has shown that RES has a wide range of pharmacological activities including antioxidant [25], anti-inflammatory [26], and inhibition of apoptosis [27]. It has been observed that resveratrol inhibits apoptosis induced by ischemia-reperfusion injury in cardiomyocytes, by upregulating the VEGF-B expression [28]. Additionally, resveratrol can also inhibit alveolar macrophage inflammatory responses, by upregulating the VEGF-B expression [23]. Therefore, for this study, RES was selected as the research target to investigate whether it exerts therapeutic effects on UVB-induced photoaging through the regulation of antioxidant, anti-inflammatory, and antiapoptotic indicators by VEGF-B, systematically elucidating the molecular mechanism of photoprotective functions of RES in UVB-induced photoaging cells and skin.

## 2. Materials and Methods

### 2.1. Reagents and Instruments

Resveratrol (RES, purity >99%), dimethyl sulfoxide (DMSO), annexinV-FITC apoptosis assay kit, superoxide dismutase (SOD) kit, glutathione (GSSH) kit, and ELISA kit for IL-6 detection were purchased from Solarbio (China). GSH and GSSH assay kits were purchased from Beyotime Biotechnology Co., Ltd. Dulbecco's Modified Eagle's Medium (DMEM), fetal bovine serum (FBS), penicillin-streptomycin solution (SP), phosphate-buffered saline (PBS), and trypsin-EDTA (0.25%) were purchased from Gibco (USA). 3-(4,5dimethylthiazol-2-yl)-2,5-diphenyltetrazolium bromide (MTT) was procured from GEN-view. TRIzol was acquired from Takara Bio (Japan). The BCA protein assay kit was provided by Thermo Fisher (China). siRNA/miRNA in vitro transfection reagents were provided by Yeasen Biotechnology Co., Ltd. (Shanghai). Lastly, siRNA VEGF-2645 and siRNA NC were procured from Sangon Biotech Co., Ltd. (Shanghai).

SH4 ultraviolet light therapy instrument with wavelength ranging between 311 and 313 nm, and having a peak wavelength of 313 nm, was purchased from Shanghai Sigma High-Tech Co., Ltd., and Corneometer CM825 skin moisture content tester was procured from CK (Germany).

### 2.2. Cell Culture

Human epidermal keratin-forming cells (Hacat) were purchased from Procell Life Sciences Co., Ltd. (Wuhan, China), with item number CL-0090. The Hacat cells were cultured in a complete medium (DMEM containing 10% FBS and 1% SP) and were stored at 37°C in a cell culture incubator maintained at 5% CO_2_ levels.

### 2.3. UVB Irradiation and Drug Administration

Hacat cells were cultured in 96-well plates at a cell density of 10^4^ cells/well and were incubated in a cell culture incubator for 24 hours. The complete medium used initially was discarded and was replaced by a fresh one containing different concentrations of RES. After 4 hours, the secondary complete medium was replaced with PBS, and the Hacat cells were irradiated with a UVB irradiation dosed at 50 mJ/cm^2^. The control group was treated to the same conditions, but without any exposure to UVB irradiation or RES treatment, whereas the model group was irradiated with UVB but did not receive RES treatment.

### 2.4. Animal Experiments

Animal experiments were carried out as previously documented, with some modifications [29]. Thirty ICR mice (male, 5-6 weeks old, weighing 18-23 g each) were purchased from Beijing Vital River Laboratory Animal Technology Co., Ltd. (license no. SCXK (Jing) 2019-0010). The mice were housed in an animal house for one week (ambient temperature range from 20 to 26°C, relative humidity of 40-70%, and 12: 12 light/dark cycle), with adequate access to food and water, to acclimatize them to the new environment. All experimental procedures, animal care, and processing were carried out in accordance with the guidelines provided in the national standard “Requirements for the Environment and Housing Facilities of Laboratory Animals” (GB 14925-2010). This experiment was approved by the “Experimental Animal Management and Experimentation” Committee of Tianjin University of Traditional Chinese Medicine. The mice were randomly divided into 3 groups: control group, model group (receiving UVB irradiation only), and RES treatment group (receiving UVB irradiation and RES administration by gavage), with 10 mice in each group. The RES being administered to the mice was dissolved in ultrapure water containing 10% ethanol and 10% Tween 80.

Intragastrical administration (2 mg/kg RES) was initiated from the second week, three times a week, wherein the control and model groups were given the same volume of saline. Following this, the back hair on each mouse was removed using an electric razor and hair removal cream, prior to the commencement of UVB irradiation treatment. Thereafter, the mice were irradiated with UVB at 40-120 mJ/cm^2^ from the fourth week onwards, and 2 mg/kg RES was administered 30 minutes prior to each irradiation. The specific weekly UVB irradiation doses are indicated in Table 1. Following the daily experiments, the skin condition of each group of mice was observed and recorded. At the end of the experiment, the mice were terminated, and blood and dorsal skin samples were collected from each mouse for subsequent analysis.

### 2.5. Cell Viability Assay

Different concentrations of RES on Hacat cells were examined by the MTT method. Hacat cells were inoculated in 96-well plates in congruence with appropriate density levels and were incubated for the desired time period. They were then washed twice with PBS. While the control group was left untreated, the model group was given 50 mJ/cm^2^ doses of UVB irradiation, and the drug administration group was given different concentrations of RES solution (0, 10, 20, 40, 60, 80, and 100 *μ*M). Hacat cells were cultured for 24 hours post and administration.. Thereafter, 10 *μ*L MTT was added to each well, and the cells were further incubated at 37°C. After 4 hours, 100 *μ*L DMSO was added to each well, and its absorbance was measured at 490 nm.

### 2.6. Measurement of SOD and GSSH

For this study, the Hacat cells were inoculated in 6-well plates at appropriate density levels and were treated according to the method listed in Section “2.3.” Following that, experiments were carried out according to the SOD and GSSH assay kit instructions to determine the activity of SOD and the content of GSSH in the cells.

### 2.7. Enzyme-Linked Immunosorbent Assay (ELISA)

For this assay, the Hacat cells were inoculated in 12-well plates at the appropriate density and were treated according to the method listed in Section “2.3.” The concentration of IL-6 was determined according to the instructions provided with the Elisa kit.

### 2.8. RNA Interference

The VEGF-B expression is silenced using siRNA. The sequences used were as follows: sense, 5′-CGACAAAGAAAUACAGAUATT (dTdT)-3′ and anti-sense, 5′-UAUCUGUAUUUCUUUGUCGTT (dTdT)-3′. Control siRNA and VEGF-B siRNA were transfected into Hacat cells according to the instructions present in the siRNA kit. After 24 hours of incubation, the culture was exposed to RES for 4 hours. Thereafter, the Hacat cells were irradiated with UVB at 50 mJ/cm^2^, and RT-qPCR experiments were performed after 4 hours.

### 2.9. Skin Image Analysis on the Backs of Mice

Water content measurements and wrinkle analysis were performed on the experimental mice once every week. The water content in each mouse was measured 3 times using a skin water content tester (CK, Germany), and the resulting data was recorded and averaged. A TIVI 700 camera was used to take pictures of the skin on the back of the mice, and the TIVI700 Analyzer software was used to analyze the skin wrinkles on the back of the mice.

### 2.10. Hematoxylin and Eosin (HE) Staining

Skin tissue from the back of ICR mice was fixed in 10% tissue fixative, embedded in paraffin, cut into 5 *μ*m sections, and stained with HE, following by the observation of histological changes under a light microscope.

### 2.11. Masson's Trichrome Stain

Paraffin sections were dewaxed in water and were then fixed in Bouin's solution, stained with celestine blue for 2-3 minutes, washed with water, stained with Mayer's hematoxylin solution for 2-3 minutes, differentiated with 1% ethanol hydrochloride for a few seconds and rinsed with water for 10 minutes, then stained with the Li chunhong acid fuchsin staining solution for 10 minutes, and rinsed with distilled water. The sections were then treated with phosphomolybdic acid solution for approximately 10 minutes. The solution at the top was decanted, and the sections were directly stained with aniline blue staining solution for 5 minutes without washing. Thereafter, the sections were treated with 0.1 ~ 0.3% glacial acetic acid solution for 2 minutes, dehydrated, and the slices sealed, to be observed under the microscope.

### 2.12. Immunohistochmeistry (IHC)

Paraffin sections were dewaxed, antigen repaired, and blocked using serum and endogenous peroxidase, primary antibodies and secondary antibodies were added, DAB color was developed, and nuclei were restained, dehydrated, and blocked further and were finally observed under the microscope.

### 2.13. Quantitative Real-Time PCR (RT-qPCR)

At the end of the treatment, cells and tissues were collected. The entire RNA was extracted using TRIzol. Post quality checks and purification, 2 *μ*g of the RNA was reverse transcribed to cDNA using a reverse transcription kit. RT-qPCR was performed using the SYBR Green Master Mix in a Bio-Rad IQ5 detection system. GADPH was used as an internal reference, and three wells were set up for each sample. The relative gene expression was calculated using the 2^-△△Ct^ method. The primer sequences used are listed in Table 2.

### 2.14. Western Blot Analysis (WB)

The Western blot technique was used to detect the protein expression in RES-treated Hacat cells and mouse skin tissue. At the end of the treatment, the cells and skin tissues that were collected were washed 3 times with PBS and placed in RIPA buffer for 30 minutes. Protein concentration was determined by the BCA Protein Assay Kit. The protein samples were subjected to SDS-PAGE gel electrophoresis and then transferred to PVDF membranes. The blots were incubated overnight at 4°C with the following primary antibodies: GADPH (AB0037, Abways Technology, 1 : 5000), p-P38MAPK (9211, CST, 1 : 1000), P38MAPK (9212, CST, 1 : 1000), COX-2 (12282, CST, 1 : 1000), nuclear NRF2 (12721, CST, 1 : 1000), p-ERK1/2 (8544, CST, 1 : 1000), ERK1/2 (4695, CST, 1 : 1000), caspase3 (9662, CST, 1 : 1000), MMP-9(13667, CST, 1 : 1000), and p-JNK (9255, CST, 1 : 1000). The membranes were visualized using a chemiluminescence analyzer. The relative density of protein bands was analyzed using the Quantity One software, and optical density values were normalized using GADPH.

### 2.15. Analysis

All data were represented in the form of mean ± standard deviation (SD) and were processed using the IBM SPSS Statistics 19.0 software. To compare the differences between multiple groups, the data was assessed using the ANOVA test followed by Dunnett's posthoc test. The values *p* < 0.05 indicate statistical significance, and *p* < 0.01 indicates a significant difference.

## 3. Results

### 3.1. Effect of RES on the Viability of UVB-Induced Photoaging Hacat Cells

**Figure 1:**
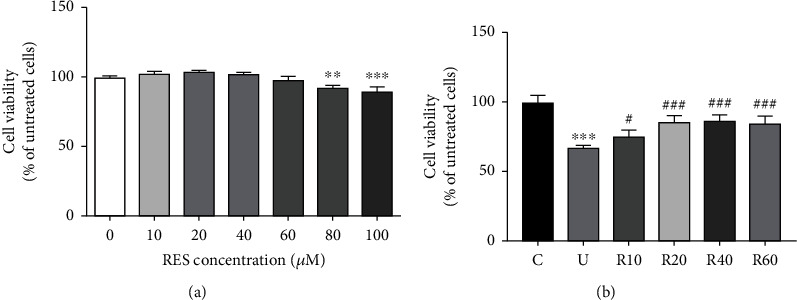
Protective effect of RES on UVB-induced photoaging Hacat cells. (a) Effect of different concentrations of RES on the viability of Hacat cells (*n* = 4). (b) Effect of RES on the viability of UVB-induced photoaging Hacat cells (*n* = 3). ^∗∗∗^*p* < 0.001, compared to the control group; #*p* < 0.05, ###*p* < 0.001, compared to the UVB group; C: control group; U: model group; R10, R20, R40, R60: RES concentration of 10, 20, 40, and 60 *μ*M drug-administered groups.

The toxic effects of different concentrations of RES on Hacat cells were examined by the MTT method. The effect of different concentrations of RES (0, 10, 20, 40, 60, 80, and 100 *μ*M) on the viability of Hacat cells was assessed. It was observed that at concentrations lesser than or equal to 60 *μ*M, RES was not cytotoxic to Hacat cells (Figure 1(a) and Table 3). Base on previous study and pre-experiments, a median lethal radiation intensity of 50 mJ/cm^2^ was selected as the subsequent dosage for UVB irradiation [30, 31]. To determine the potential protective effect of RES on Hacat cells, we assayed Hacat cells post-UVB irradiation treatment using the MTT method. As shown in Figure 1(b) and Table 4, the cell viability of Hacat cells was significantly reduced after exposure to UVB, and RES at concentrations of 10, 20, 40, and 60 *μ*M increased the viability of Hacat cells by 7.89, 18.30, 19.45, and 17.14%, respectively, compared to the model group.

### 3.2. Effect of RES on UVB-Induced Photoaging in the Skin of ICR Mice

**Figure 2:**
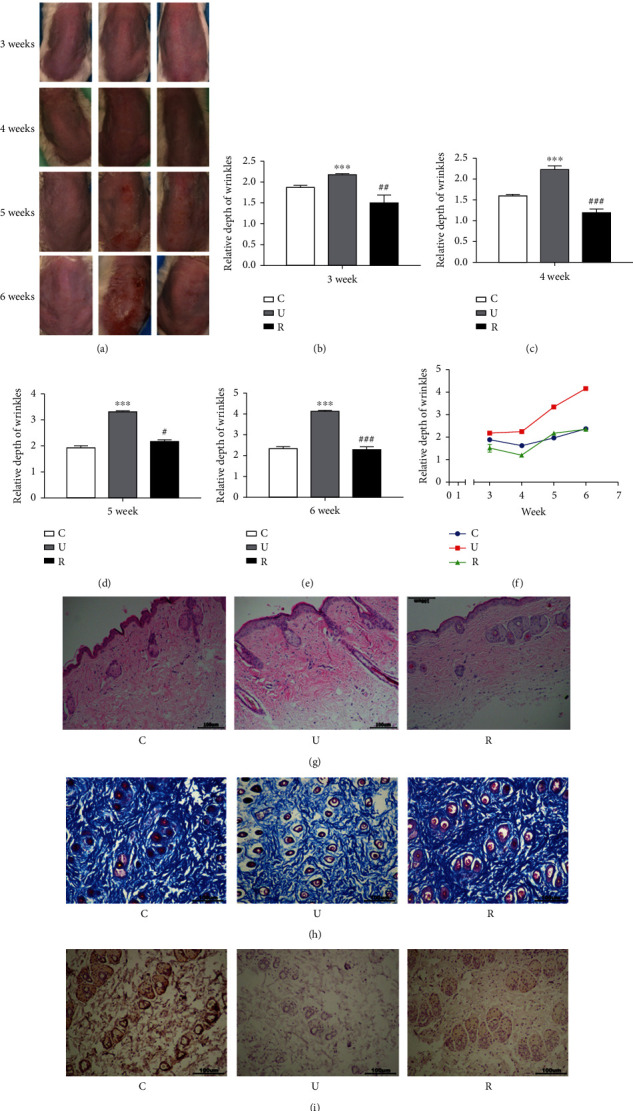
Effect of RES on the skin of UVB-induced photoaging ICR mice. (a) Effect of RES on skin surface morphology in UVB-induced photoaging ICR mice (*n* = 6). (b)–(f) The depth of skin wrinkles in ICR mice measured weekly from week 3 onwards (*n* = 6). (g) Histopathological changes in the skin of each group of ICR mice (*n* = 6, scale bar = 100 *μ*M). (h) Content of collagen fibers in the skin of each group of ICR mice (*n* = 6, scale bar = 100 *μ*M). (i) Collagen III content in the skin of ICR mice in each group (*n* = 6, scale bar = 100 *μ*M). ^∗∗∗^*p*≦0.001, compared with the control group; #*p* < 0.05, ##*p* < 0.01, ###*p* < 0.001, compared with the model group. C: control group; U: model group; R: RES drug-administered group.

The effects of RES on skin morphology, water content and wrinkle depth, pathological changes, and collagen deposition in mice were examined individually. As shown in Figures 2(a)–(f) and Table 5, as the experiment progressed, the skin of the model mice gradually became rough and hypertrophic, with the presence of erythema, peeling, ulceration, and large deep wrinkles, as compared to the control group. Moreover, compared to the model group, the RES-administered group also did not show any distinct sign of erythema, crusting, or the presence of large deep wrinkles, and the skin surface was similar to that of the control group. As shown in Table 6, UVB irradiation of the dorsal skin of the ICR mice resulted in a significant decrease in skin water content, while RES gavage administration in the ICR mice resulted in a significant increase in skin water content.

Histological analysis of the skin was carried out by HE staining. As depicted in Figure 2(g), the model group showed marked levels of hyperkeratosis with an incidence of hyperkeratosis in the epidermis, as compared to the control group, with a large infiltration of inflammatory cells and a scattered and fractured arrangement of collagen fibers in the dermis. However, it was observed that the epidermal structural disorder had improved in the drug-administered group as compared to the model group. The effect of RES on collagen fibril damage in the dermis of ICR mice was assessed by Masson trichrome staining. As shown in Figure 2(h), compared to the control group, the model group showed a significant decrease in collagen fibers stained with the blue dye in the dermis, indicating that UVB irradiation can lead to collagen fiber destruction. However, the reduction of collagen fibers in the dermis of ICR mice was significantly countered in the drug-administered group, as compared to the model group, suggesting that RES can prevent UVB-induced destruction of collagen fibers in the skin of ICR mice. The effect of RES on the damage to collagen fibers in the skin of ICR mice was further illustrated by the assessment of type III collagen by IHC. As shown in Figure 2(i), in the control group, a significant type III collagen immunoreactivity was observed in the dermis of the skin of ICR mice. Compared to the control group, type III collagen immunoreactivity was significantly reduced throughout the dermis in the model group, indicating that UVB irradiation caused a decrease in collagen content in the skin of ICR mice. On the other hand, compared to the model group, type III collagen immunoreactivity in the dermis of the drug-administered group was significantly greater, indicating that RES alleviated the loss of collagen in the skin of ICR mice.

### 3.3. Effect of RES on UVB-Induced Photoaging Cells and Skin Apoptosis

**Figure 3:**
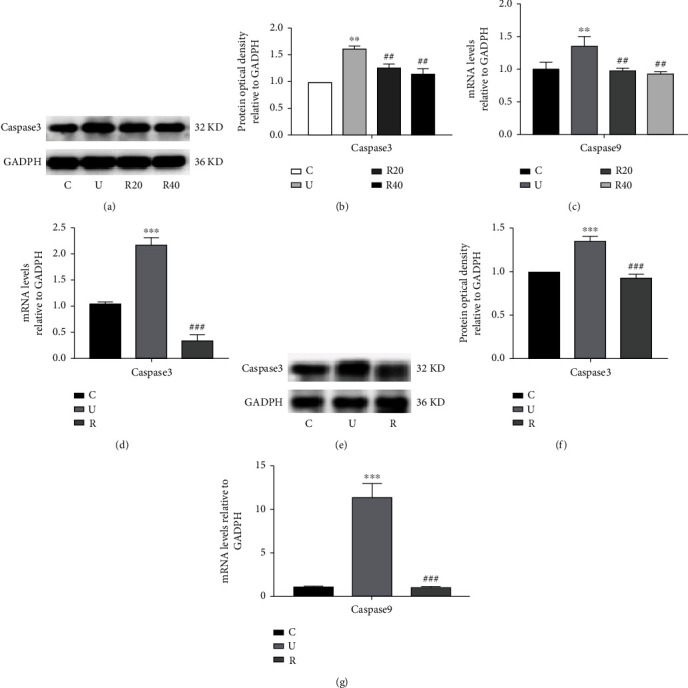
Effect of RES on apoptosis in UVB-induced photoaging Hacat cells and ICR mouse skin. (a) Changes in caspase3 protein levels in photoaging Hacat cells (*n* = 3). (b) Relative grey values of caspase3 protein in photoaging Hacat cells using GADPH as an internal reference. (c) Relative expression content of caspase9 mRNA in photoaged Hacat cells using GADPH as an internal reference (*n* = 3). (d) Relative expression content of mRNA of caspase3 in the skin of photoaging ICR mice using GADPH as an internal reference (*n* = 6). (e) Changes in caspase3 protein levels in the skin of photoaging ICR mice (*n* = 6). (f) Relative grey values of caspase3 protein in the skin of photoaging ICR mice using GADPH as an internal reference. (g) Relative expression content of mRNA of caspase9 in the skin of photoaging ICR mice using GADPH as an internal reference (*n* = 6). ^∗∗^*p* < 0.01, ^∗∗∗^*p* < 0.001, compared to the control group; ##*p* < 0.01, ###*p* < 0.001, compared to the model group. C: control group; U: model group; R20, R40: RES concentration of 20 and 40 *μ*M drug-administered groups.

To investigate the mechanism of inhibition of apoptosis by RES, we examined the relevant genes and proteins in the photoaging Hacat cells using RT-qPCR and WB assays. As shown in Figures 3(a) and 3(b) and Table 7, the expression of caspase3 protein was significantly higher in the model group as compared to the control group. The expression of caspase3 protein was also significantly lower in the drug-administered group as compared to the model group. As shown in Figures 3(c) and Table 8, UVB irradiation significantly increased the expression of caspase9 mRNA in Hacat cells, whereas the expression of caspase9 mRNA decreased significantly after the cells were given 20 *μ*M and 40 *μ*M of RES.

To further verify the mechanism of anti-apoptotic function of RES, in vivo, we examined the relevant genes and proteins in the skin of photoaging ICR mice using RT-qPCR and WB assays. As shown in Figures 3(d) and 3(g) and Tables 9 and 10, UVB irradiation of ICR mice significantly promoted caspase3 and caspase9 in their skin (*p* < 0.001), while RES gavage significantly decreased its expression (*p* < 0.001). The results of WB experiments were consistent with those of RT-qPCR experiments as well and are shown in Figures 3(e) and 3(f) and Table 11, respectively.

### 3.4. Effect of RES on Gene Expression of MMPs in UVB-Induced Photoaging Cells and Skin

**Figure 4:**
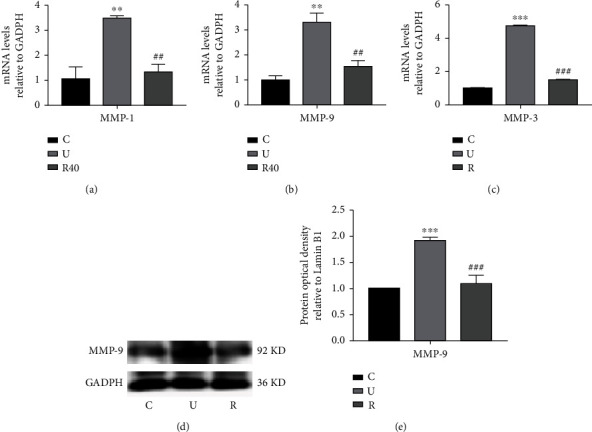
Effect of RES on the gene expression of MMPs in UVB-induced photoaging Hacat cells and ICR mouse skin. (a) Relative expression content of MMP-1mRNA in photoaged Hacat cells using GADPH as an internal reference (*n* = 3). (b) Relative expression content of MMP-9mRNA in photoaging Hacat cells with GADPH as an internal reference (*n* = 3). (c) Relative expression content of MMP-3mRNA in the skin of photoaged ICR mice using GADPH as an internal reference (*n* = 6). (d) Changes in MMP-9 protein levels in the skin of photoaged ICR mice (*n* = 6). (e) Relative grayscale values of MMP-9 protein in the skin of photoaged ICR mice using GADPH as an internal reference. ^∗∗^*p* < 0.01, ^∗∗∗^*p* < 0.001, compared to the control group; ##*p* < 0.01, ###*p* < 0.001, compared to the model group. C: control group; U: model group; R40: RES concentration of 40 *μ*M drug-administered group.

To elucidate the molecular mechanism through which RES protects Hacat cells from UVB-induced damage, the mRNA levels of MMP-1 and MMP-9 were measured by RT-qPCR post-UVB irradiation of the cells. The results are shown in Figures 4(a) and 4(b) and Table 12. UVB irradiation significantly increased the expression of MMP-1 and MMP-9 mRNA in Hacat cells, whereas the expression of MMP-1 and MMP-9 mRNA decreased significantly after the cells were given 40 *μ*M of RES. Meanwhile, the differences in the expression of MMPs in the skin of ICR mice between different groups were investigated by RT-qPCR and WB experiments. As shown in Figures 4(c)–(e) and Tables 13 and 14, the expression of MMP-3 and MMP-9 in the skin of ICR mice in the model group showed a significant increase. Moreover, levels of MMP-3 and MMP-9 in the skin of ICR mice in the drug-administered (RES) group were lower than that in the model group, indicating that RES decreases the expression of MMP-3 and MMP-9 in the skin of ICR mice (*p* < 0.001), thereby alleviating UVB-induced degradation of the extracellular matrix in the skin of photodamaged mice.

### 3.5. Effect of RES on Antioxidant-Related Indicators in UVB-Induced Photoaging Hacat Cells and ICR Mice

**Figure 5:**
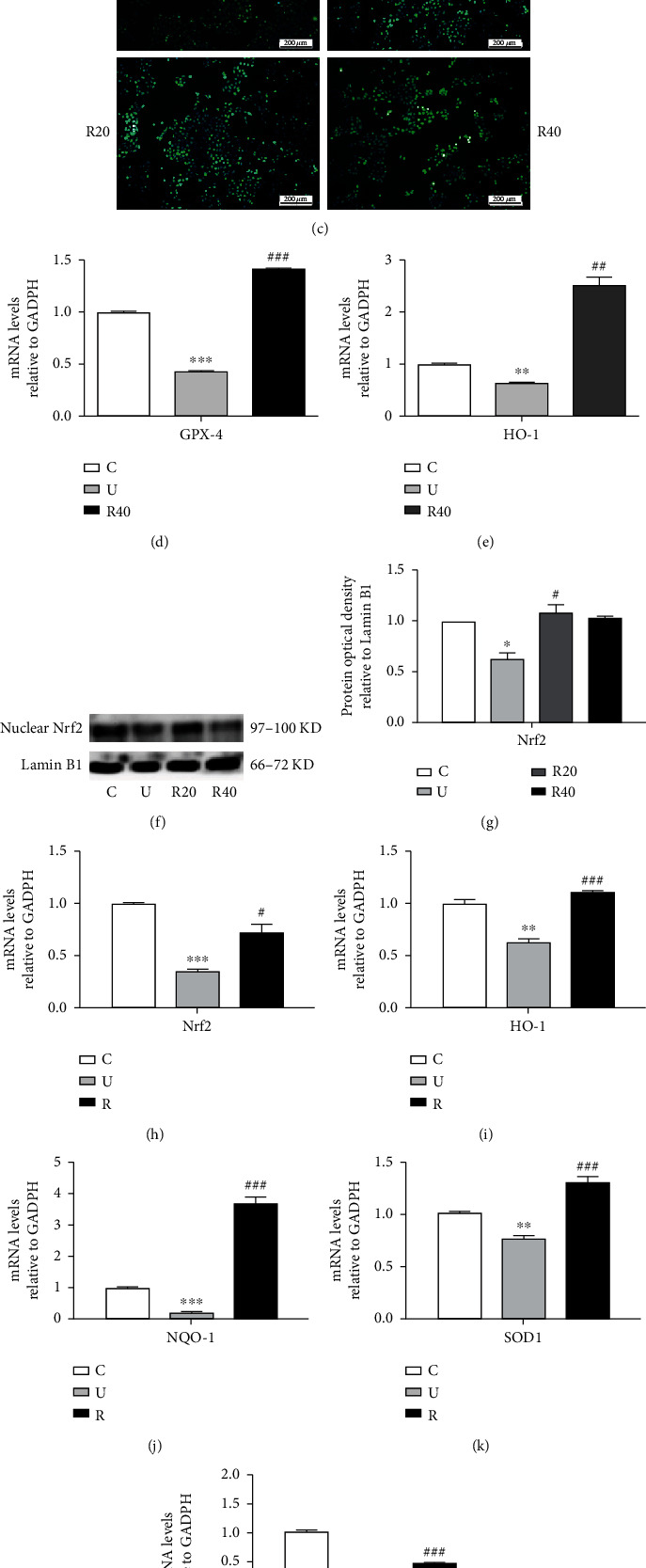
Effect of RES on antioxidant-related indicators in the skin of UVB-induced photoaging Hacat cells and ICR mice. (a) Changes in GSSH content in photoaging Hacat cells (*n* = 3). (b) Changes in SOD content in photoaging Hacat cells (*n* = 3). (c) Changes in ROS content in photoaging Hacat cells (*n* = 3, scale bar: 200 *μ*M). (d) Relative expression content of GPX-4mRNA in photoaging Hacat cells using GADPH as an internal reference (*n* = 3). (e) Relative expression content of HO-1mRNA in photoaging Hacat cells using GADPH as an internal reference (*n* = 3). (f) Changes in nuclear Nrf2 protein levels in photoaging Hacat cells (*n* = 3). (g) Relative grey values of intranuclear Nrf2 protein in photoaging Hacat cells using Lamin B1 as an internal reference. (h) Relative mRNA expression of Nrf2 in the skin of photoaging ICR mice using GADPH as an internal reference (*n* = 6). (i) Relative mRNA expression of HO-1 in the skin of photoaging ICR mice using GADPH as an internal reference (*n* = 6). (j) Relative mRNA expression of NQO1 in the skin of photoaging ICR mice using GADPH as an internal reference (*n* = 6). (k) Relative mRNA expression of SOD1 in the skin of photoaging ICR mice using GADPH as an internal reference (*n* = 6). (l) Relative mRNA expression of GPX-4 in the skin of photoaging ICR mice using GADPH as an internal reference (*n* = 6). ^∗^*p* < 0.05, ^∗∗^*p* < 0.01, ^∗∗∗^*p* < 0.001, compared to the control group; #*p* < 0.05, ##*p* < 0.01, ###*p* < 0.001, compared to the model group. C: control group; U: model group; R20, R40: RES concentration 20 and 40 *μ*M drug-administered groups.

To demonstrate that the protection of Hacat cells from UVB irradiation by RES is related to its antioxidant effect, the GSSH content, SOD activity, and ROS levels were measured using kits, mRNA levels of GPX-4 and HO-1 were measured through RT-qPCR assays, and the expression levels of Nrf2 protein in the nucleus were measured by the WB assay, once the cells were irradiated with UVB. As shown in Figures 5(a) and 5(b) and Tables 15 and 16, the GSSH and SOD levels of Hacat cells were significantly decreased after UVB irradiation, and in the RES drug-administered group, both GSSH and SOD levels were significantly increased. As shown in Figure 5(c), the ROS content of Hacat cells significantly increased after UVB irradiation, whereas ROS activity decreased at both 20 and 40 *μ*M RES concentrations. As shown in Figures 4(d) and 4(e) and Table 17, the mRNA levels of GPX-4 and HO-1 were significantly decreased in Hacat cells after UVB irradiation, while the administration of RES significantly increased GPX-4 and HO-1 expression. As shown in Figures 5(f) and 5(g) and Table 18, the levels of nuclear Nrf2 protein in Hacat cells were significantly decreased after UVB irradiation; however, RES increased the expression of nuclear Nrf2 protein. These results suggest that RES can exert its protective effect by regulating the intracellular oxidative system.

To further verify the in vivo antioxidant activity of RES, the expression levels of Nrf2 and its phase II detoxification enzyme genes in the skin of ICR mice between different groups were examined by the RT-qPCR assay. As shown in Figures 5(h)–5(l) and Table 19, the mRNA levels of Nrf2, HO-1, NQO1, SOD1, and GPX-4 were significantly reduced in the model group as compared to the control group, with GPX-4 mRNA exhibiting the sharpest reduction in its levels. Compared to the model group, the mRNA of each gene was significantly increased in the drug-administered group, with the most increase in NQO1 mRNA. This suggests that RES can reduce UVB-induced oxidative stress in the skin of ICR mice by affecting the gene expression of Nrf2 and its phase II detoxification enzymes, with RES having a stronger effect on the downstream genes of the antioxidant pathway than its upstream genes.

### 3.6. Effect of RES on Inflammation-Related Indicators in UVB-Induced Photoaging Hacat Cells and ICR Mice

**Figure 6:**
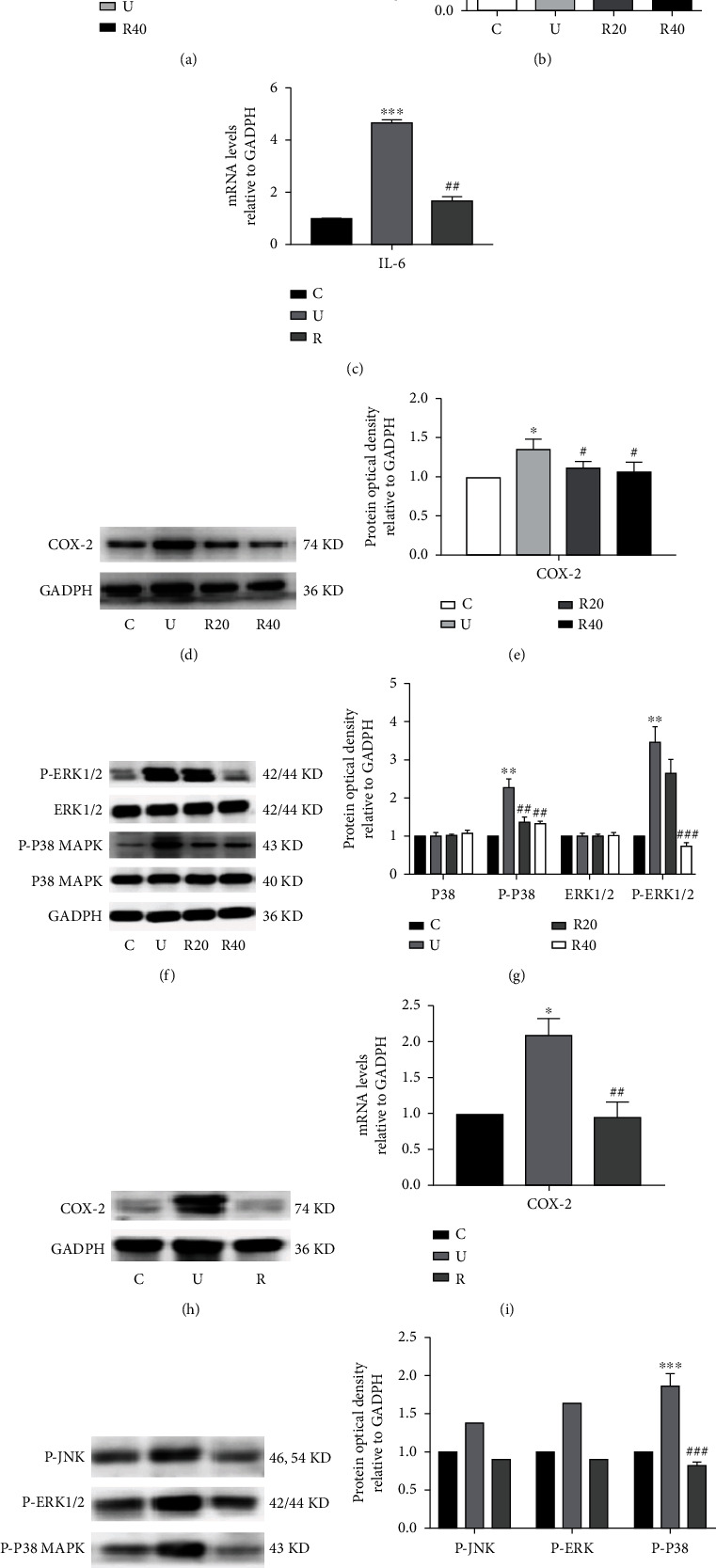
Effect of RES on inflammation-related indicators in the skin of UVB-induced photoaging Hacat cells and ICR mice. (a) Relative expression content of mRNA for IL-6 and TNF-*α* in photoaging Hacat cells using GADPH as an internal reference (*n* = 3). (b) Changes in IL-6 content in photoaging Hacat cells (*n* = 3). (c) Relative expression content of mRNA of IL-6 in the skin of photoaging ICR mice using GADPH as an internal reference (*n* = 6). (d) Changes in COX-2 protein levels in photoaging Hacat cells (*n* = 3). (e) Relative grey values of COX-2 protein in photoaging Hacat cells using GADPH as an internal reference.(f) Changes in P-ERK1/2, ERK1/2, P-P38MAPK, and P38MAPK protein levels in photoaging Hacat cells (*n* = 3). (g) Relative grey values of P-ERK1/2, ERK1/2, P-P38MAPK, and P38MAPK proteins using GADPH as an internal reference. (h) Changes in COX-2 protein levels in the skin of photoaging ICR mice (*n* = 6). (i) Relative grayscale values of COX-2 proteins using GADPH as an internal reference. (j) Changes in P-JNK, P-ERK1/2, and P-P38MAPK protein levels in the skin of photoaging ICR mice (*n* = 6).(k) Relative grey values of P-JNK, P-ERK1/2, and P-P38MAPK proteins in the skin of photoaging ICR mice using GADPH as an internal reference. ^∗^*p* < 0.05, ^∗∗^*p* < 0.01, ^∗∗∗^*p* < 0.001, compared to control; #*p* < 0.05, ##*p* < 0.01, ###*p* < 0.001, compared to model. C: control group; U: model group; R20, R40: RES concentration of 20 and 40 *μ*M drug-administered groups.

The cytokines IL-6 and TNF-*α* play an important role in inflammation [32], so, we examined their mRNA expression levels in Hacat cells.  Figure 6(a) and Table 20 show that the mRNA levels of inflammatory cytokines IL-6 and TNF-*α* were significantly upregulated in Hacat cells post-UVB exposure, whereas the mRNA levels of IL-6 and TNF-*α* were significantly lower when the RES concentration was 40 *μ*M. To further verify the effect of RES on the inflammatory cytokine IL-6, ELISA assays were performed, the results of which are shown in Figure 6(b) and Table 21, whereby the level of the inflammatory cytokine IL-6 significantly increased after the cells were exposed to UVB, and when the concentration of RES was 20 and 40 *μ*M, the level of IL-6 reduced significantly. Subsequently, the effect of RES on IL-6 in the skin of ICR mice was also examined. As shown in Figure 6(c) and Table 22, the expression of IL-6 in the skin of ICR mice in the model group was found to be elevated, whereas the expression of IL-6 in the RES drug-administered group was significantly reduced, attenuating the inflammatory response induced by UVB irradiation.

Additionally, the COX-2 signaling pathway and MAPK pathway-related proteins play a major role in the production of proinflammatory cytokines in response to UV irradiation [33]. Therefore, the expression levels of COX-2 protein and MAPK pathway-related proteins were also examined, through WB experiments. The results, as presented in Figures 6(d)–(g) and Tables 23 and 24, showed that the expression levels of COX-2 protein and MAPK pathway-related proteins increased significantly after being exposed to UVB, whereas their expression levels decreased significantly in the RES group. Moreover, as shown in Figures 6(h)–(k) and Tables 25 and 26, COX-2 and MAPK pathway-related proteins exhibited lower levels of expression in the skin of control ICR mice and exhibited higher levels of expression in the model group, with a significant difference in expression levels (*p* < 0.05). ICR mice administered with RES effectively reduced their expression levels of COX-2 and MAPK pathway-related proteins, indicating that RES has anti-inflammatory effects that are exerted through the COX-2 pathway and MAPK pathway-related pathways.

### 3.7. RES Exerts Photoaging Protection through Upregulation of VEFG-B Expression

**Figure 7:**
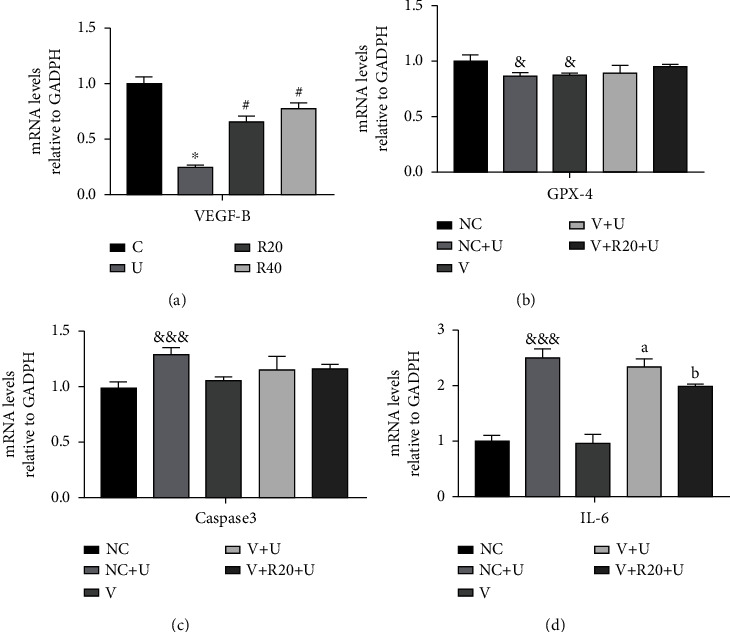
Effect of VEGF-B on the photoprotective effect of RES. (a) Effect of transfected siRNA on the expression of mRNA of VEGF-B in Hacat cells (*n* = 3). (b) Effect of transfected siRNA on the expression of mRNA for GPX-4 in Hacat cells (*n* = 3). (c) Effect of transfected siRNA on the expression of mRNA for caspase3 in Hacat cells (*n* = 3). (d) Effect of transfected siRNA on the expression of mRNA for IL-6 in Hacat cells (*n* = 3). ^∗^*p* < 0.05, compared to the control group; #*p* < 0.05, compared to the model group; &*p* < 0.05, &&&*p* < 0.001, compared to the NC group; (a)) *p* < 0.001, compared to the V group; (b)) *p* < 0.05, compared to the V + U group. C: control group; U: model group; R20, R40: RES concentration of 20 and 40 *μ*M drug-administered groups. NC group: transfected siRNA NC, also negative transfection control group; NC + U group: transfected siRNA NC followed by UVB irradiation; V group: transfected siRNA VEGF-B; V + U group: transfected siRNA VEGF-B followed by UVB irradiation; V + R20 + U group: transfected siRNA VEGF-B followed by 20 *μ*M RES drug-administration, 4 hours post-UVB irradiation.

To verify the potential pharmacological effects of RES on VEGF-B as a target, RT-qPCR experiments were performed to detect the mRNA expression levels of VEGF-B under different conditions. As seen in Figure 7(a) and Table 27, UVB irradiation significantly reduced the mRNA expression of VEGF-B in the model group as compared to the control group, whereas in RES-pretreated Hacat cells, the inhibition of mRNA expression of VEGF-B was improved and its expression increased significantly, as compared to the model group. To confirm the antioxidant, antiapoptotic, and anti-inflammatory activity of RES through the targeting of VEGF-B, three indicators, GPX-4, caspase3, and IL-6, were detected using the RT-qPCR assay. As presented in Figures 7(b)–(d) and Table 28, the mRNA expression levels of GPX-4 reduced in the NC + U group as compared to the NC group, but caspase3 and IL-6 expression increased significantly. Compared to the V + U group, for the V + R20 + U group, the mRNA expression levels of GPX-4 were elevated, but the mRNA expression levels of caspase3 were almost unchanged, and the mRNA expression levels of IL-6 were significantly lower. This suggests that RES treats photoaging through the antioxidant and antiapoptotic effects by targeting the VEGF-B.

## 4. Discussion

RES is a polyphenolic compound widely found in various plants such as grape skin, *Veratrum nigrum* L., and *Reynoutria japonica* Houtt. [34], but its role and the mechanism involved in preventing or delaying photoaging have not yet been researched exhaustively. In the present study, we found that RES pretreatment exerted a combined effect on the treatment of UVB-induced photoaging through four pathways: anti-MMP expression, anti-inflammatory, antiapoptotic, and antioxidant activities, in vivo and in vitro.

In this study, we found that RES was nontoxic to Hacat cells at concentrations less than or equal to 60 *μ*M, and that RES at concentrations of 10, 20, 40, and 60 *μ*M positively influenced the reduction in cell viability of UVB photoaging Hacat cells, promoting cell proliferation and exerting a protective effect as a result of it. To explore the mechanism of inhibition of apoptosis by RES in vivo and vitro, we used WB and RT-qPCR assay to detect caspase3 protein expression and the mRNA expression level of caspase9 and found that RES pretreatment reduced caspase3 protein expression and the mRNA expression level of caspase9 both in vivo and in vitro. Additionally, we also found that RES pretreatment reduced the mRNA expression level of caspase3 in the skin of photoaging mice, by RT-qPCR. All these suggest that RES has an antiapoptotic effect, and that its effect on apoptosis is manifested through the basic mechanism of inhibition of heterologous activation of caspase (caspase3) and homologous activation of caspase (caspase9).

The most prominent feature of photoaging is the synthesis and degradation of collagen, and both UVB irradiation and excess ROS can induce the production of MMPs [35], which are responsible for degrading collagen. MMPs mainly include MMP-1, MMP-3, and MMP-9 [36], of which MMP-1 is the main protease that degrades collagen; MMP-3 degrades types III and IV collagen and gelatin and activates MMP-1 and other members of the MMP family, and MMP-9 is the main protease that degrades gelatin. We examined the mRNA expression levels of MMP-1 and MMP-9 in Hacat cells, and MMP-3 in the skin of photoaging mice by RT-qPCR, and observed that RES preadministration reduced the mRNA expression levels of MMP-1, MMP-3, and MMP-9, and that RES preadministration reduced the expression of MMP-9 in photoaging mice as well, as ascertained through WB experiments. This indicates that RES preadministration can alleviate the degradation of extracellular matrix in UVB-induced photoaging cells and mice skin.

To prevent skin damage caused by UV-induced excessive levels of ROS and to regulate epidermal homeostasis, skin cells activate their endogenous defense system and exert their antioxidant function [37]. The results of our experiments showed that RES preadministration balanced intracellular oxidative stress by increasing GSSH levels and enhancing SOD activity, as well as by reducing ROS levels, while promoting the expression of mRNA for GPX-4 and HO-1 and the nuclear Nrf2 protein, and by promoting the expression of mRNA for Nrf2 and its downstream genes HO-1, NQO1, SOD1, and GPX-4, balancing oxidative stress in the skin of photoaged mice. This suggests that RES can exert its protective effects by modulating the oxidative system within photoaged Hacat cells and mice skin cells.

TNF-*α* is a widely distributed proinflammatory factor in the body and mediates various physiological and biochemical responses such as inflammation, apoptosis, and immune response [38] and further promotes the synthesis of the cellular inflammatory factor IL-6. The COX-2 signaling pathway plays a major role in the production of proinflammatory cytokines in response to UVB irradiation. Experimental results show that RES preadministration reduces the inflammation induced by UVB irradiation, in vivo and in vitro, by inhibiting the expression of the inflammatory factors IL-6 and TNF-*α*, and COX-2 proteins.

Through WB experiments, we determined that RES preadministration reduced P-ERK1/2 and P-P38MAPK protein levels in Hacat cells, but had no effect on ERK1/2 and P38MAPK protein expression. We also examined the protein expression of MAPK pathway proteins P-JNK, P-ERK1/2, and P-P38MAPK in the skin of photoaging mice and found the results to be the same as that for the cells. This indicates that RES may exert its protective effect on photoaging Hacat cells and ICR mice by inhibiting the phosphorylation of MAPK pathway proteins.

The mRNA expression levels of GPX-4 and caspase3 in Hacat cells were affected by RES through the targeting of VEGF-B, balancing intracellular oxidative stress and inhibiting apoptosis and demonstrating its antioxidant and antiapoptotic effects in the process. Meanwhile, the results showed that RES did not affect the mRNA expression levels of IL-6 in Hacat cells by targeting VEGF-B, and its anti-inflammatory effects were manifested through other targets.

**Figure 8:**
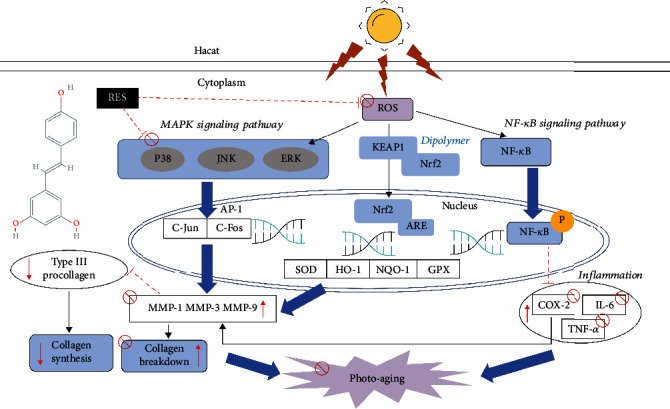
Mechanism of photoprotection by RES against UVB-induced photoaging.

In conclusion, RES has a protective effect on UVB-induced photoaging in Hacat cells and ICR mice, and its mechanism of action may include reducing the expression of MMPs and secretion of collagen and inflammatory factors by inhibiting ROS-mediated MAPK and COX-2 signaling pathways, balancing of oxidative stress in cells and mouse skin by promoting Nrf2 signaling pathway, and inducing antiapoptotic effects through the inhibition of caspase activation; RES also exerts its antioxidant and antiapoptotic effects by targeting the VEGF-B. Therefore, the photoprotective properties of RES can alleviate the photoaging induced by UVB irradiation. The mechanism of photoprotection by RES against UVB-induced photoaging was shown in Figure 8.

## Figures and Tables

**Table 1:** Specific weekly UVB irradiation doses.

Week	UVB irradiation dose
1	—
2	—
3	—
4	40 mJ/cm^2^
5	80 mJ/cm^2^
6	120 mJ/cm^2^
7	120 mJ/cm^2^

**Table 2:** RT-qPCR primer sequences.

Gene		Forward primer(5′-3′)	Reverse primer (3′-5′)
Human	GPX-4	GAGGCAAGACCGAAGTAAACTAC	CCGAACTGGTTACACGGGAA
	HO-1	AAGACTGCGTTCCTGCTCAAC	AAAGCCCTACAGCAACTGTCG
	IL-6	ACTCACCTCTTCAGAACGAATTG	CCATCTTTGGAAGGTTCAGGTTG
	TNF-*α*	CCTCTCTCTAATCAGCCCTCTG	GAGGACCTGGGAGTAGATGAG
	MMP-1	AAAATTACACGCCAGATTTGCC	GGTGTGACATTACTCCAGAGTTG
	MMP-9	TGTACCGCTATGGTTACACTCG	GGCAGGGACAGTTGCTTCT
	VEGF-B	GAGATGTCCCTGGAAGAACACA	GAGTGGGATGGGTGATGTCAG
	GADPH	GGAGCGAGATCCCTCCAAAAT	GGCTGTTGTCATACTTCTCATGG
Mouse	GPX-4	TGTGCATCCCGCGATGATT	CCCTGTACTTATCCAGGCAGA
	SOD1	AACCAGTTGTGTTGTCAGGAC	CCACCATGTTTCTTAGAGTGAGG
	Nrf2	CTTTAGTCAGCGACAGAAGGAC	AGGCATCTTGTTTGGGAATGTG
	HO-1	AGGTACACATCCAAGCCGAGA	CATCACCAGCTTAAAGCCTTCT
	NQO1	AGGATGGGAGGTACTCGAATC	TGCTAGAGATGACTCGGAAGG
	IL-6	CTGCAAGAGACTTCCATCCAG	AGTGGTAGACAGGTCTGTTGG
	Caspase3	CTCGCTCTGGTACGGATGTG	TCCCATAAATGACCCCTTCATCA
	Caspase9	GGCTGTTAAACCCCTAGACCA	TGACGGGTCCAGCTTCACTA
	MMP-3	GGCCTGGAACAGTCTTGGC	TGTCCATCGTTCATCATCGTCA
	GADPH	AGGTCGGTGTGAACGGATTTG	GGGGTCGTTGATGGCAACA

**Table 3:** Effect of different concentrations of RES on the viability of Hacat cells (*n* = 4).

RES concentration	0	10	20	40	60	80	100
Cell viability	100.00	102.41	103.78	102.18	97.92	92.74^∗∗^	89.78^∗∗∗^

^∗∗^ *p* < 0.01, ^∗∗∗^*p* < 0.001, compared to the control group.

**Table 4:** Effect of RES on the viability of UVB-induced photoaging Hacat cells (*n* = 3).

	C	U	R10	R20	R40	R60
Cellviability	100.00	68.14^∗∗∗^	76.03^#^	86.44^###^	87.59^###^	85.28^###^

^∗∗∗^ *p* < 0.001, compared to the control group; #*p* < 0.05, ###*p* < 0.001, compared to the UVB group; C: control group; U: model group; R10, R20, R40, R60: RES concentration of 10, 20, 40, and 60 *μ*M drug-administered groups.

**Table 5:** The depth of skin wrinkles in ICR mice measured weekly from week 3 onwards (*n* = 6).

	Third week	Fourth week	Fifth week	Sixth week
C	1.90	1.62	1.97	2.38
U	2.19^∗∗∗^	2.25^∗∗∗^	3.35^∗∗∗^	4.16^∗∗∗^
R	1.51^##^	1.20^###^	2.18^#^	2.35^###^

^∗∗∗^ *p*≦0.001, compared with the control group; #*p* < 0.05, ##*p* < 0.01, ###*p* < 0.001, compared with the model group. C: control group; U: model group; R: RES drug-administered group.

**Table 6:** Water content in the skin of each group of ICR mice measured weekly from week 2 onwards (*n* = 6).

	Second week	Third week	Fourth week	Fifth week	Sixth week
C	35.95 ± 1.57	35.64 ± 1.71	33.82 ± 0.94	30.43 ± 1.11	34.97 ± 0.58
U	34.28 ± 1.49	27.04 ± 1.81^∗∗∗^	23.08 ± 1.59^∗∗∗^	21.22 ± 0.77^∗∗∗^	21.50 ± 1.29^∗∗∗^
R	34.80 ± 0.99	30.76 ± 0.56^#^	33.35 ± 0.16^##^	32.67 ± 0.94^###^	35.20 ± 0.58^###^

^∗∗∗^ *p* < 0.001, compared to controlgroup; #*p* < 0.05, ##*p* < 0.01, ###*p* < 0.001, compared to the model group. C: control group; U: model group; R: RES drug-administered group.

**Table 7:** Relative grey values of caspase3 protein in photoaging Hacat cells using GADPH as an internal reference (*n* = 3).

	C	U	R20	R40
Caspase3	1.00	1.62^∗∗^	1.27^##^	1.14^##^

^∗∗^ *p* < 0.01, compared to the control group; ##*p* < 0.01, compared to the model group. C: control group; U: model group; R20, R40: RES concentration of 20 and 40 *μ*M drug-administered groups.

**Table 8:** Relative expression content of caspase9 mRNA in photoaged Hacat cells using GADPH as an internal reference (*n* = 3).

	C	U	R20	R40
Caspase9	1.00	1.35^∗∗^	0.99^##^	0.94^##^

^∗∗^ *p* < 0.01, compared to the control group; ##*p* < 0.01, compared to the model group. C: control group; U: model group; R20, R40: RES concentration of 20 and 40 *μ*M drug-administered groups.

**Table 9:** Relative expression content of mRNA of caspase3 in the skin of photoaging ICR mice using GADPH as an internal reference (*n* = 6).

	C	U	R
Caspase3	1.05	2.18^∗∗∗^	0.35^###^

^∗∗∗^ *p* < 0.001, compared to the control group; ###*p* < 0.001, compared to the model group. C: control group; U: model group; R: RES drug-administered group.

**Table 10:** Relative expression content of mRNA of caspase9 in the skin of photoaging ICR mice using GADPH as an internal reference (*n* = 6).

	C	U	R
Caspase9	1.07	11.40^∗∗∗^	1.10^###^

^∗∗∗^ *p* < 0.001, compared to the control group; ###*p* < 0.001, compared to the model group. C: control group; U: model group; R: RES drug-administered group.

**Table 11:** Relative grey values of caspase3 protein in the skin of photoaging ICR mice using GADPH as an internal reference (*n* = 6).

	C	U	R
Caspase3	1.00	1.36^∗∗∗^	0.94^###^

^∗∗∗^ *p* < 0.001, compared to the control group; ###*p* < 0.001, compared to the model group. C: control group; U: model group; R: RES drug-administered group.

**Table 12:** Relative expression content of MMP-1and MMP-9 mRNA in photoaged Hacat cells using GADPH as an internal reference (*n* = 3).

	C	U	R40
MMP-1	1.06	3.52^∗∗^	1.36^##^
MMP-9	1.01	3.36^∗∗^	1.59^##^

^∗∗^ *p* < 0.01, compared to the control group; ##*p* < 0.01, compared to the model group. C: control group; U: model group; R40: RES concentration of 40 *μ*M drug-administered group.

**Table 13:** Relative expression content of MMP-3 mRNA in the skin of photoaged ICR mice using GADPH as an internal reference (*n* = 6).

	C	U	R
MMP-3	1.00	4.80^∗∗∗^	1.54^###^

^∗∗∗^ *p* < 0.001, compared to the control group; ###*p* < 0.001, compared to the model group. C: control group; U: model group; R: RES drug-administered group.

**Table 14:** Relative grayscale values of MMP-9 protein in the skin of photoaged ICR mice using GADPH as an internal reference (*n* = 6).

	C	U	R
MMP-9	1.00	1.93^∗∗∗^	1.10^###^

^∗∗∗^ *p* < 0.001, compared to the control group; ###*p* < 0.001, compared to the model group. C: control group; U: model group; R: RES drug-administered group.

**Table 15:** Changes in GSSH content in photoaging Hacat cells (*n* = 3).

	C	U	R20	R40
GSSH content	1.85	1.42^∗∗∗^	1.82^###^	1.73^###^

^∗∗∗^ *p* < 0.001, compared to the control group; ###*p* < 0.001, compared to the model group. C: control group; U: model group; R20, R40: RES concentration 20 and 40 *μ*M drug-administered groups.

**Table 16:** Changes in SOD content in photoaging Hacat cells (*n* = 3).

	U	R20	R40
SOD content	10.19	10.66	11.53^###^

###*p* < 0.001, compared to the model group. U: model group; R20, R40: RES concentration 20 and 40 *μ*M drug-administered groups.

**Table 17:** Relative expression content of GPX-4 and HO-1 mRNA in photoaging Hacat cells using GADPH as an internal reference (*n* = 3).

	C	U	R40
GPX-4	1.00	0.44^∗∗∗^	1.42^###^
HO-1	1.00	0.65^∗∗^	2.55^##^

^∗∗^ *p* < 0.01, ^∗∗∗^*p* < 0.001, compared to control group; ##*p* < 0.01, ###*p* < 0.001, compared to model group. C: control group; U: model group; R40: RES concentration 40 *μ*M drug-administered groups.

**Table 18:** Relative grey values of intranuclear Nrf2 protein in photoaging Hacat cells using Lamin B1 as an internal reference (*n* = 3).

	C	U	R20	R40
Nrf2	1.00	0.63^∗^	1.09^#^	1.03

^∗^ *p* < 0.05, compared to the control group; #*p* < 0.05, compared to the model group. C: control group; U: model group; R20, R40: RES concentration 20 and 40 *μ*M drug-administered groups.

**Table 19:** Relative mRNA expression of Nrf2, HO-1,NQO1, SOD1, and GPX-4 in the skin of photoaging ICR mice using GADPH as an internal reference (*n* = 6).

	C	U	R
Nrf2	1.01	0.36^∗∗∗^	0.72^#^
HO-1	1.00	0.64^∗∗^	1.11^###^
NQO1	1.00	0.23^∗∗∗^	3.66^###^
SOD1	1.02	0.77^∗∗^	1.30^###^
GPX-4	1.02	0.02^∗∗∗^	0.48^###^

^∗∗^ *p* < 0.01, ^∗∗∗^*p* < 0.001, compared to the control group; #*p* < 0.05, ###*p* < 0.001, compared to the model group. C: control group; U: model group; R: RES drug-administered group.

**Table 20:** Relative expression content of mRNA for IL-6 and TNF-*α* in photoaging Hacat cells using GADPH as an internal reference (*n* = 3).

	C	U	R40
IL-6	1.00	35.43^∗∗∗^	4.16^###^
TNF-*α*	1.02	4.47^∗∗∗^	3.36^###^

^∗∗∗^ *p* < 0.001, compared to control; ###*p* < 0.001, compared to model group. C: control group; U: model group; R40: RES concentration of 40 *μ*M drug-administered group.

**Table 21:** Changes in IL-6 content in photoaging Hacat cells (*n* = 3).

	C	U	R20	R40
IL-6 content	1.10	1.40^∗∗^	0.96^##^	0.21^###^

^∗∗^ *p* < 0.01, compared to the control group; ##*p* < 0.01, ###*p* < 0.001, compared to the model group. C: control group; U: model group; R20, R40: RES concentration of 20 and 40 *μ*M drug-administered groups.

**Table 22:** Relative expression content of mRNA of IL-6 in the skin of photoaging ICR mice using GADPH as an internal reference (*n* = 6).

	C	U	R
IL-6	1.01	4.71^∗∗∗^	1.71^##^

^∗∗∗^ *p* < 0.001, compared to control group; ##*p* < 0.01, compared to model group. C: control group; U: model group; R: RES drug-administered group.

**Table 23:** Relative grey values of COX-2 protein in photoaging Hacat cells using GADPH as an internal reference (*n* = 3).

	C	U	R20	R40
COX-2	1.00	1.37^∗^	1.12^#^	1.06^#^

^∗^ *p* < 0.05, compared to the control group; #*p* < 0.05, compared to the model group. C: control; U: model group; R20, R40: RES concentration of 20 and 40 *μ*M drug-administered groups.

**Table 24:** Relative grey values of P-ERK1/2, ERK1/2, P-P38MAPK, and P38MAPK proteins in photoaging Hacat cells using GADPH as an internal reference (*n* = 3).

	C	U	R20	R40
P38	1.00	1.02	1.04	1.10
P-P38	1.00	2.29^∗∗^	1.40^##^	1.34^##^
ERK1/2	1.00	1.01	1.02	1.04
P-ERK1/2	1.00	3.46^∗∗^	2.67	0.76^###^

^∗∗^ *p* < 0.01, compared to the control group; ##*p* < 0.01, ###*p* < 0.001, compared to the model group. C: control group; U: model; R20, R40: RES concentration of 20 and 40 *μ*M drug-administered groups.

**Table 25:** Relative grayscale values of COX-2 proteins in the skin of photoaging ICR mice using GADPH as an internal reference (*n* = 6).

	C	U	R
COX-2	1.00	2.10^∗^	0.96^##^

^∗^ *p* < 0.05, compared to the control group; ##*p* < 0.01, compared to the model group. C: control group; U: model group; R: RES drug-administered group.

**Table 26:** Relative grey values of P-JNK, P-ERK1/2, and P-P38MAPK proteins in the skin of photoaging ICR mice using GADPH as an internal reference (*n* = 6).

	C	U	R
P-JNK	1.00	1.40	0.92
P-ERK	1.00	1.66	0.90
P-P38	1.00	1.89^∗∗∗^	0.83^###^

^∗∗∗^ *p* < 0.001, compared to the control group; ###*p* < 0.001, compared to the model group. C: control group; U: model group; R: RES drug-administered group.

**Table 27:** Effect of transfected siRNA on the expression of mRNA of VEGF-B in Hacat cells (*n* = 3).

	C	U	R20	R40
VEGF-B	1.00	0.26^∗^	0.67^#^	0.78^#^

^∗^ *p* < 0.05, compared to the control group; #*p* < 0.05, compared to the model group. C: control group; U: model group; R20, R40: RES concentration of 20 and 40 *μ*M drug-administered groups.

**Table 28:** Effect of transfected siRNA on the expression of mRNA for GPX-4, caspase3, and IL-6 in Hacat cells (*n* = 3).

	NC	NC + U	V	V + U	V + R20 + U
GPX-4	1.00	0.88^&^	0.88^&^	0.90	0.96
Caspase3	0.99	1.30^&&&^	1.06	1.17	1.17
IL-6	1.00	2.52^&&&^	0.98	2.36^a^	2.01^b^

&*p* < 0.05, &&&*p* < 0.001, compared to the NC group; ^a^*p* < 0.001, compared to the V group; ^b^*p* < 0.05, compared to the V + U group. NC group: transfected siRNA NC, also negative transfection control group; NC + U group: transfected siRNA NC followed by UVB irradiation; V group: transfected siRNA VEGF-B; V + U group: transfected siRNA VEGF-B followed by UVB irradiation; V + R20 + U group: transfected siRNA VEGF-B followed by 20 *μ*M RES drug-administration, 4 hours post-UVB irradiation.

## Data Availability

The data that supports the findings of this study are available in the supplementary material of this article.
